# Study of a Steel’s Energy Absorption System for Heavy Quadricycles and Nonlinear Explicit Dynamic Analysis of its Behavior under Impact by FEM

**DOI:** 10.3390/ma8105345

**Published:** 2015-10-10

**Authors:** José Ángel López Campos, Abraham Segade Robleda, José Antonio Vilán Vilán, Paulino José García Nieto, Javier Blanco Cordero

**Affiliations:** 1 Department of Mechanical Engineering, University of Vigo, Vigo 36203, Spain; segade.vigo@gmail.com (J.A.L.C.); asegade@uvigo.es (A.S.R.); jvilan@uvigo.es (J.A.V.V.); javierblancordero@gmail.com (J.B.C.); 2 Department of Mathematics, University of Oviedo, Faculty of Sciences, C/Calvo Sotelo s/n, Oviedo 33007, Spain

**Keywords:** steel longitudinal energy absorption system, impact analysis, explicit dynamic analysis, finite element modelling, vehicle crashworthiness, quadricycles

## Abstract

Current knowledge of the behavior of heavy quadricycles under impact is still very poor. One of the most significant causes is the lack of energy absorption in the vehicle frame or its steel chassis structure. For this reason, special steels (with yield stresses equal to or greater than 350 MPa) are commonly used in the automotive industry due to their great strain hardening properties along the plastic zone, which allows good energy absorption under impact. This paper presents a proposal for a steel quadricycle energy absorption system which meets the percentages of energy absorption for conventional vehicles systems. This proposal is validated by explicit dynamics simulation, which will define the whole problem mathematically and verify behavior under impact at speeds of 40 km/h and 56 km/h using the finite element method (FEM). One of the main consequences of this study is that this FEM–based methodology can tackle high nonlinear problems like this one with success, avoiding the need to carry out experimental tests, with consequent economical savings since experimental tests are very expensive. Finally, the conclusions from this innovative research work are given.

## 1. Introduction

A vehicle frame, also known as its chassis, is the main supporting structure of a motor vehicle to which all other components are attached, and it is comparable to the skeleton of an organism. Until the 1930s, virtually every motor vehicle had a structural frame, separate from the car’s body. This construction design is known as *body-on-frame*. Since then, nearly all passenger cars have received unibody construction, meaning their chassis and bodywork have been integrated into one another. Typically the material used to construct vehicle chassis and frames is carbon steel. In the case of a separate chassis, the frame is made up of structural elements called the rails or beams. These are ordinarily made of steel channel sections, constructed by folding, rolling or pressing steel plates. In this way, special steels (with yield stress equal to or greater than 350 MPa) are commonly used for the rail components in the automotive industry due to their great strain hardening properties along the plastic zone, which allows good energy absorption under impact [1].

In recent years, the use of small motorized vehicles has increased to become a real alternative to conventional vehicles in certain situations. However, one of the existing drawbacks for manufacturers is that the regulating legislation varies greatly between countries.

The Japanese government is a pioneer at legislating these small-scale vehicles. In fact, there is a vehicle category called *kei car* (mini car) whose requirements were first published in 1949 (maximum length of 2.8 m and maximum width of 1 m) and have evolved over time so that today they can be a maximum of 3.4 m long and 1.48 m wide, and a have a maximum displacement of 660 cm^3^. In November 2012, of 79.87 million vehicles in Japan, 35.4% were mini cars and mini trucks [2].

In the European Union, such vehicles are known by the generic name of quadricycles and there has been a new regulation for category L vehicles [3] since 2013. This regulation classifies as light quadricycles (L6e) those that have an unladen mass equal to or less than 350 kg with a maximum speed limit of 45 km/h; heavy quadricycles (L7e) are those that have an unladen mass equal to or less than 450 kg and an engine power of below 15 kW instead of a maximum speed. In this second category, there are commercial models that can reach 100 km/h.

With respect to the safety requirements for quadricycles, technically in Europe these vehicles are not required to fulfill the stringent safety controls applied to conventional passenger vehicles, although they are governed by the same physical laws when there is an impact. Japan, at the forefront again, established in 2007 that the offset deformable barrier test should also be performed on the mini car, at a test speed of 56 km/h [2].

The first published statistical reference on the consequences of a quadricycle impact is an Austrian study [4] into vehicle accidents (specifically, category L6e). It concluded that the number of fatalities per vehicle was nearly three times greater for a quadricycle than for a passenger car, and the number of fatalities per accident injury was almost nine times greater for lightweight vehicles than for passenger cars. This statistic describes the danger of such vehicles and the fact that although the maximum speed of a quadricycle is lower than a conventional vehicle, when there is a head-on collision with a conventional vehicle, the small vehicle undergoes much greater accelerations than in a collision with rigid barriers at full speed.

In short, this innovative paper is organized as follows: firstly, Section 2 describes energy absorption systems for conventional vehicles, Section 3 looks at minicar impact performance, Section 4 presents the proposed energy absorption system, and Section 5 discusess the FEM explicit dynamic validation of the proposed system, with the results and conclusions of this research work being found in Section 6 and Section 7.

## 2. Longitudinal Energy Absorption in Conventional Vehicles

Passive safety comes into play once an accident is inevitable and its function is to reduce as far as possible the fatal consequences of an impact. On impact, the structure of a vehicle has two main functions:

- The first function is to absorb the kinetic energy of the vehicle while keeping those allowable decelerations for the survival of occupants.
- The second function is to preserve the integrity of the passenger compartment and so avoid the intrusion of rigid components into it.

### 2.1. Energy Absortion Systems

**Figure 1:**
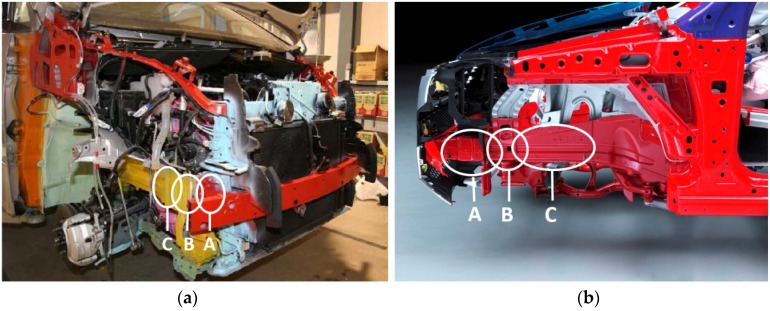
The structure of a passenger van (**a**) and a passenger car (**b**).

Studying the structural behavior of a vehicle in the event of longitudinal impact reveals that the structure of a vehicle is composed of components whose function is to deform in a programmed way under impact (deformable parts), *i.e.*, to absorb energy. These components in first instance are the vehicle bumper, and the initial section of the front rails of the vehicle (see Figure 1) [5]. In most conventional vehicles the front rail is made up of two parts: a replaceable piece called the crash box (A), which folds in a controlled pattern under impact, and a second part, the frame rail, with an initial section (B) which also folds in a controlled pattern and a second section (C) which transmits the force to the vehicle structure.

The ideal behavior of the front structure of a vehicle would provide a constant resistance to deformation (while not reaching the passenger safety cell). This would cause a constant force producing deceleration that is not only great but which must also be bearable by the occupants. Various studies focus on the behavior of the axial crushing of tubes [6,7], where folding patterns of different geometries are studied, and the evolution of compressive force versus displacement is tested. The study of geometry buckling initiators [8] is also very common. These are small predeformations in the tube that will achieve a reduction in the initial peak force for the collapse of the tube. The use of buckling initiators is very common in crash boxes and in the initial section of frame rails in order to reduce the initial buckling force .

### 2.2. Energy Absorption Distribution between the Different Components of the Front Structure of an Automobile

**Figure 2:**
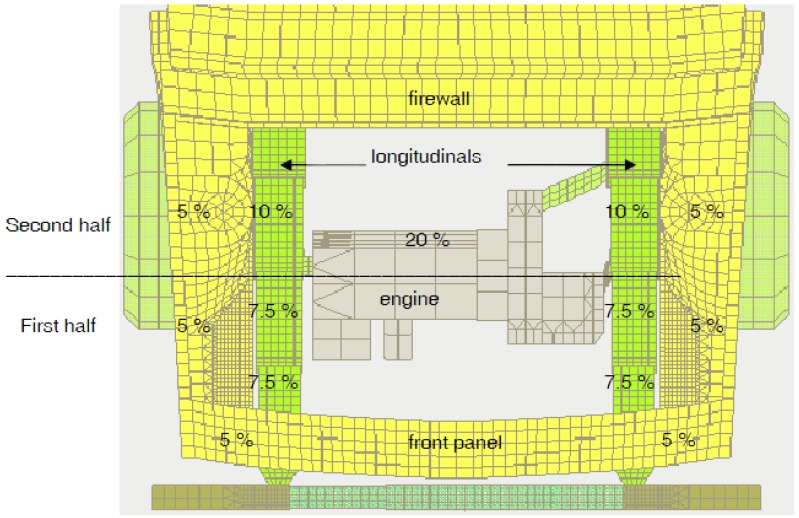
Estimated energy absorption percentages in the frontal structure of an automobile.

The distribution of energy absorption between different longitudinal components can be studied. Several works have studied this, particularly doctoral theses [9,10,11], which make a proposal for the division of energy for an impact at 56 km/h against a rigid wall as shown in Figure 2.

Figure 2 shows a top view of the front of a car, where the distribution of energy absorption can be seen. The front panel of the vehicle absorbs only 10% of the total energy, and the components which absorb more energy are the front rail of the car with 50% of the energy, followed by the motor, which absorbs 20%. Within each of the two front rail components, 7.5% of the total energy is absorbed by the crash box (Figure 1A), 7.5% by the front section of the frame rail (Figure 1B) and another 10% by the frame rail (Figure 1C). These energy absorption percentages would be different depending on the impact speed. If the velocity is not high, the components which are in the first half of the structure would be able to absorb all the kinetic energy, without deformation of the second half. These percentages correspond to an impact that does not involve deformation of the passenger compartment and the impacted object is a rigid wall which does not absorb energy.

## 3. Impact Performance of Minicars

Now that the impact performance for conventional vehicles has been shown, we will focus on the performance for quadricycles and minicars. The literature on the impact crashworthiness of minicars has been mostly written relatively recently and in all events is very scarce. One of the most complete references is the study by Hardy [12], a European study of L category vehicles (unladen mass under 350 kg or 450 kg) and whether they could meet the same regulatory requirements as M1 cars (conventional cars). The study shows wide disparity between the results of frontal-impact tests for the quadricycles reviewed, and indicates that no current quadricycle would comply with M1 category safety requirements, which suggests that the evolution of safety in quadricycles still has a long way to go.

Moreover, EuroNCAP (European New Car Assessment Programme) undertook a safety campaign for heavy quadricycles (L7e) [13] in 2014, which tested the following models: Renault Twizy 80, Ligier IXO JS Line 4 Places, Tazzari Zero and Club Car Villager 2+2 LSV. The study results indicate that “all of the quadricycles tested showed critical safety problems”, and according to the executive management of ETSC (European Transport Safety Council) “these vehicles already satisfy a minimum set of requirements which is clearly not enough as the tests show” [13].

Mizuno [2] produced a work on passive safety for minicars, which clearly states that their security is less than that of a conventional vehicle due to the technological challenge of their small size and mass. The work concludes that the lack of safety is mainly due to the lack of space, and that a lower mass implies larger decelerations, and it recommends that this type of vehicle have a force limiter and pre-tensioner system for the seat belt and shrinkable columns for the steering because intrusions into the passenger compartment are one of the main problems with this type of vehicle.

**Figure 3:**
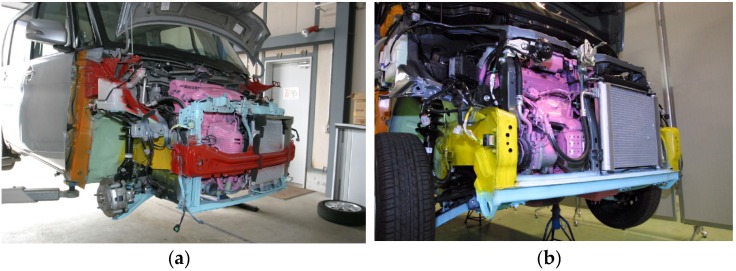
The structure of two minicars (**a**) and (**b**).

In a way, the chassis structure of a minicar is similar to that of a conventional vehicle, with the handicap of having less space available. Figure 3 shows the chassis structure of two minicars whose impact performance was tested [5]. Minicar type A (see Figure 3a) has two front rails connected directly to the crossbeam that supports the bumper, while minicar type B (see Figure 3b) does not have a crossbeam for the bumper and front rails are joined together by a horizontal beam over the suspension.

In these two vehicles it can be seen that there is a lack of the energy absorbing components in the longitudinal direction that are usually mounted in conventional vehicles (crash box, A in Figure 1b, or buckling initiators at the frame rail B in Figure 1b).

When there is a head-on collision, the kinetic energy of the occupant must be absorbed by the strain energy of the vehicle and, moreover, by the vehicle restraint system. The distribution of absorbed energy that corresponds to each of these factors can be studied. In a conventional vehicle the energy absorbed by the vehicle corresponds to 30%–65% of the initial kinetic energy, while for a quadricycle it would correspond to 20%–35% [2]. This makes it necessary to use a pretensioner system for the seat belt because the restraint system must absorb most of the energy. 

An important conclusion about the structure of the minicar is its poor energy absorption through the strain energy of the vehicle. The objective of this work is to improve the percentage of strain energy absorbed by the structure of the vehicle by implementing absorbing systems in its chassis design.

## 4. Proposed Energy Absorbing System

Having examined the poor energy absorption of the quadricycle structure, we propose a design for a front rail formed by a crash box and a deformable frame rail that meets the absorption percentage existing in conventional vehicles (see Figure 2 above) [9,14,15]:

- 7.5% of the initial kinetic energy is absorbed by the crash box;
- 7.5% of the initial kinetic energy is absorbed by the initial section of the frame rail;
- 10% of the initial kinetic energy is absorbed by the final section of the frame rail.

### 4.1. Proposed Model of the Front Rail

**Figure 4:**
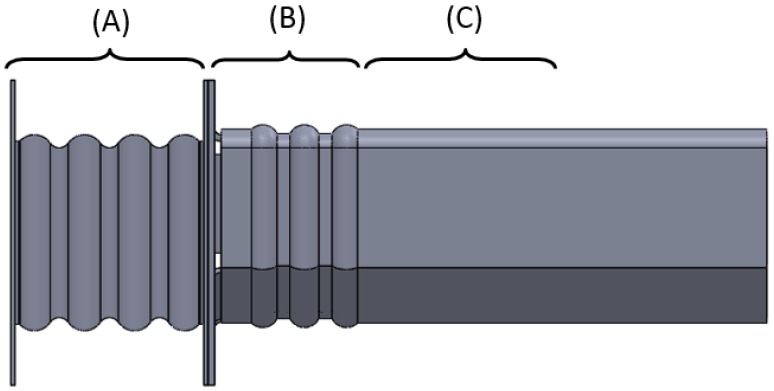
Parts of the proposed front rail.

The initial objective is to propose a model of front rail for a quadricycle (see Figure 4). This will consist of an initial part that will be the crash box (A), a second part formed by the frame rail, with an initial variable section for a buckling initiator (B), followed by a second section (C) with a basic geometry. A flange is used to adapt the geometry of the crash box to the frame rail. According to Witteman [9], approximately 7.5% of the initial kinetic energy is absorbed by the crash box, another 7.5% by the initial part of the frame rail and 10% by the second section. These percentages of energy absorption are relative to the initial kinetic energy of a vehicle travelling at 56 km/h which crashes against a rigid wall.

### 4.2. Three–Dimensional Model

**Figure 5:**
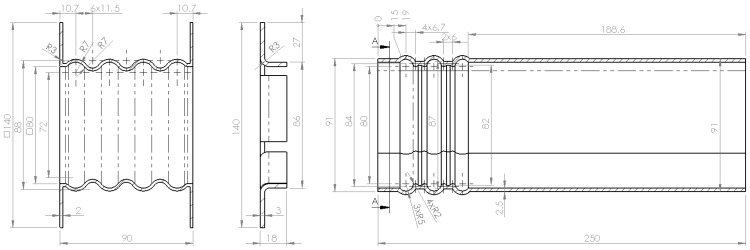
Side view of crash box, flange and frame rail.

All the components are made from metal sheet the thickness of which varies as follows: 2 mm thick for the crash box, 2.5 mm thick for the frame rail and 3 mm thick for the flange which joins both together (see Figure 5). The thicknesses of each component are decided after FEM simulations are carried out. Furthermore, the percentages of energy absorbed must comply with the requirements established by Witteman [9]: the crash box must have a lower thickness to ensure that it is the first component that deforms. In relation to the specific geometry, the crash box was made with a typical wavy geometry and the frame rail was also made with a first section that deforms more easily and has wavy geometry. The crash box is formed by an undulating surface with a 7 mm radius, and a distance between centers of 11.5 mm and a total length of 86 mm. The thickness is formed outward of the initial surface. The frame rail also has an initial undulating surface with rounded outer forms of 5 mm in radius, and double rounding at the inner part of 2 mm in radius with a distance between centers of 6 mm covering a length of 62 mm.

**Figure 6:**
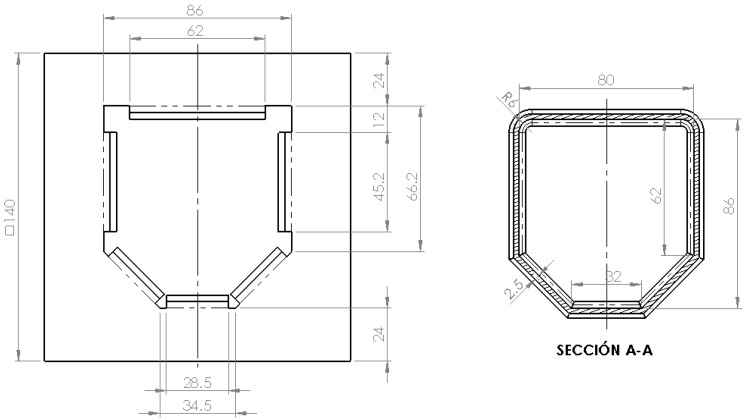
Frontal view of flange and frame rail.

Along the cross section, the crash box has a fully square cross section and the frame rail has a non-regular hexagonal section shown in Figure 6.

**Figure 7:**
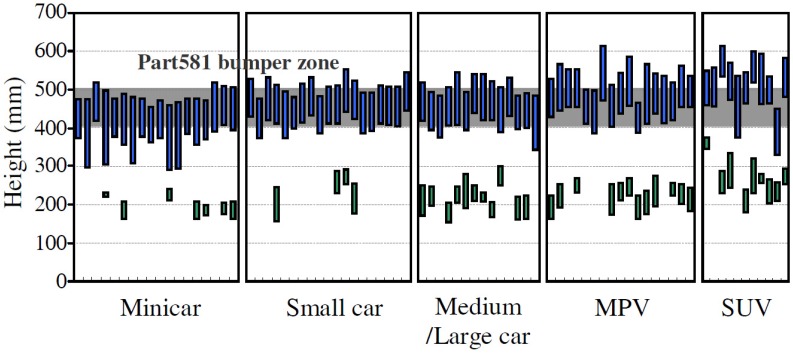
The ground clearance of front rails (blue bars) and subframes (green bars).

The irregular hexagonal cross section for the frame rail causes the rigidity of the upper half of the section to be greater than the rigidity of its lower half. When there is an impact at an intermediate position, the deformation is larger at the bottom than at the top, so bending at the lower part would occur and light rising of the front could be achieved. For quadricycles a more rigid upper part is beneficial because under impact the shorter car tends to slide under the taller one. Bumpers of minicars are usually lower than those of conventional vehicles (see Figure 7). The height difference is usually between 100 and 200 mm and it is further accentuated when compared with the height of the Multi-Purpose Vehicles (MPV) and Sport-Utility Vehicles (SUV).

### 4.3. Layout Analysis of the Proposed Energy Absorption System

With respect to the layout analysis, it is really important both to the dimensional study and to the study of mass of the energy absorption system proposed, since the regulations for minicars andquadricycles are very different depending on the country. The proposed design focuses on the quadricycles according to European regulations. Therefore, its limitation is its mass. In the proposed case, the mass of the crash box, flanges and first section of the frame rail means a mass increase of about 4.5 kg, representing about 1% of the unladen mass of heavy quadricycles. This increased mass of 1% is considered very small compared to the benefits obtained when this energy absorption system is mounted in the vehicle.

From the dimensional point of view, this system means an additional length of 160 mm for the minicars without a crossbeam for the bumper (see Figure 3b), and 100 mm for those with a crossbeam (see Figure 3a). For example, this dimensional variation in relation to the maximum dimensions of the minicar in Japan (3.4 m) implies less than 5% and 3%, respectively.

## 5. Validation of the Proposed Geometry

Once the geometry of the front rail components is defined, an explicit dynamic FEM simulation will be conducted in order to study the full performance of the system. This simulation is prepared considering a heavy quadricycle category L7e, with unladen mass not exceeding 450 kg. Batteries in case of electric vehicles, or fuel for others, and occupants should be added to that mass. The impact will be performed at 56 km/h to try to reproduce the performance of a conventional vehicle according to Witteman [9].

Preprocessing will be done with Ansys [16], defining mesh, boundary, initial conditions and interactions. The material model is defined in a dynamic explicit preprocessor (Ls-PrePost), defining the behavior of both static and dynamic models. The complete system will be solved using Ls-Dyna and post-processing also will be done with Ls-PrePost [16].

### 5.1. Geometrical Definition of the Performed Simulation Test

**Figure 8:**
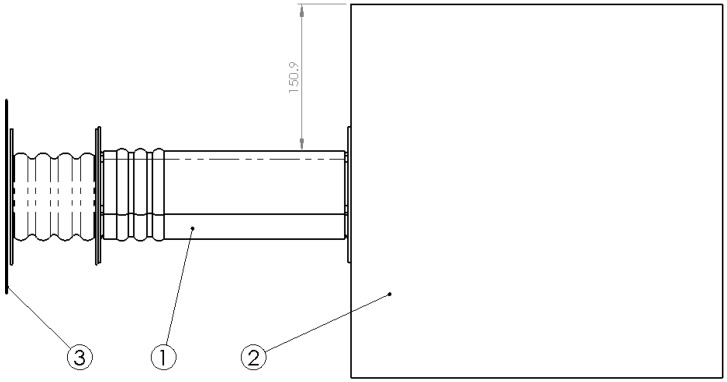
Side view of wall, front rail, ballast assembly.

The geometry of the simulation test is shown in Figure 8. The front rail geometry is the one previously defined (see Subsection 4.2). A ballast of 450 kg is placed at the back of the frame rail. This will simulate the mass of the minicar and is tied to the frame rail by a flange, which adapts the geometry from one to the other. In the simulation the ballast will work as an inertial mass, so the mass of parts situated before the front rail must be deducted. Heavy quadricyles have an unladen mass equal to or less than 450 kg, so the mass of the occupants and fuel (batteries if it is electric) must be added. Therefore, a mass of 450 kg is considered a reasonable value, and it is defined using a cube of 385 mm along each side. The impact is made against a 200 × 200 mm rigid wall (3) that is two millimeters thick.

The height of the center of gravity of the ballast will coincide with the centroid of the hexagonal cross section in order to minimize torques on the ballast. In this case, the distance between the upper face of the frame rail and the ballast is 150.9 mm (see Figure 8).

### 5.2. Mesh and Boundary Conditions

Boundary conditions are established according to the defined test. In this case, the impact wall is a rigid component whose nodes are restricted to six degrees of freedom. Moreover, the assembly formed by the crash box, frame rail, plus 450 kg of ballast is defined with an initial speed of 56 km/h. The goal of the simulation is to study the energy absorption by the components and the effects of different variables such as the impact speed.

All metal parts are generated as surfaces. The whole 3D model must be consistent, indicating the thickness towards the top and bottom of the CAD surface and must be checked to ensure that there is no interference between elements at the initial time. In this case, contact zones between parts are created so that the primary surface of both parts is the same, and thicknesses are assigned towards opposite sides. In those areas where contact between parts does not occur on the primary surface, a mesh connection is generated to ensure contact. In the case under study, this occurs in the contact between the crash box and the flange from the frame rail, where the original surfaces are separated from each other by a distance of 3 mm, which is filled when the flange thickness is assigned.

**Figure 9:**
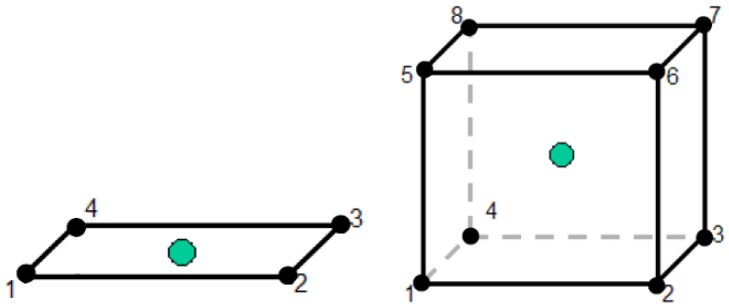
Two-dimensional and three-dimensional elements for meshing.

Coherently with the type of geometry, surfaces are meshed using SHELL elements (see Figure 9) [16]; more specifically, 4-node-linear-quadrilateral elements with reduced linear integration.The “Mapped Face Meshing” tool is employed giving a size of 2 mm to the crash box and frame rail, and 3.5 mm to the flanges. Rigid components (wall and ballast) are meshed with SOLID elements (see Figure 9), *i.e.*, 8-node-hexahedron elements with reduced integration [16].

Contacts between the different metal components (crash box, flange and frame rail) are defined as bonded, as is the contact between the ballast and the posterior flange. Body interactions are enabled to detect collisions between components throughout the evolution of the problem over time.

### 5.3. Mathematical Model Used for Dynamic Explicit Solution

The basic equations solved by an explicit dynamic analysis express the conservation of mass, momentum and energy in Lagrange coordinates. These, together with a material model and a set of initial and boundary conditions, define the complete solution of the problem. For Lagrange formulations, the mesh moves and distorts with the material it models, so conservation of mass is automatically satisfied. For each time step, these equations are solved explicitly for each element in the model, based on values input at the end of the previous time step. Only mass and momentum conservations are enforced. However, in well-posed explicit simulations, mass, momentum and energy should all be conserved.

Summarizing, here a highly nonlinear geometrical and material nonlinearity generally occurs. Therefore, we can write the equation of motion in the discretized system as (in terms of the discretization nodal parameter a) [17,18,19]:

(1) $$M\left(a\right)\ddot{a}+C\left(a\right)\dot{a}+P\left(a\right)+f=0$$

where:

- M(a) is the diagonal mass matrix, depending on a if nonlinearity exists;
- $\ddot{a}$ are the components of nodal acceleration;
- C(a) is the damping matrix, depending on a if nonlinearity exists;
- $\dot{a}$ are the components of nodal velocity;
- P(a)≡Ka is the vector of resisting internal forces, where *K* is the stiffness matrix;
- f is the vector of external forces.

The *explicit dynamics solver* uses a central difference time integration scheme. The semi-discrete equations of motion at time *n* are [17,18,19]:

(2) $$M{\ddot{a}}^{n}=P^{n}-F^{n}+H^{n}$$

where:

- ${\ddot{a}}^{n}$ are the components of nodal acceleration at time *n*;
- $P^{n}$ are the external and body forces at time *n*;
- $F^{n}$ is the stress divergence vector at time *n*;
- $H^{n}$ is the hourglass resistance at time *n*.

with the accelerations at time $n-\frac{1}{2}$ determined, the velocities at time $n+\frac{1}{2}$ at direction *i* (i=1,2,3) are found from [16,17,18,19]:

(3) $${\dot{a}}_{i}^{n+\frac{1}{2}}={\dot{a}}_{i}^{n-\frac{1}{2}}+{\ddot{a}}_{i}^{n}\Delta t^{n}$$

Finally, the positions are updated to time n+1 by integrating the velocities:

(4) $$a_{i}^{n+1}=a_{i}^{n}+{\dot{a}}_{i}^{n+\frac{1}{2}}\Delta t^{n+\frac{1}{2}}$$

Some advantages of using this method for time integration for nonlinear problems are as follows:

- The equations become uncoupled and can be solved directly (explicitly). There is no requirement for iteration during time integration;
- No convergence checks are needed since the equations are uncoupled;
- No inversion of the stiffness matrix is required. All nonlinearities (including contact) are included in the internal force vector.

Another two important issues are the stability time step and mass scaling. Indeed:

- To ensure stability and accuracy of the solution, the size of the time step used in Explicit time integration is limited by the Courant-Friedrichs-Levy (CFL) condition [20];
- This condition implies that the time step be limited such that a disturbance (stress wave) cannot travel further than the smallest characteristic element dimension in the mesh, in a single time step;
- Thus the time step criteria for solution stability is as follows:
  (5) $$\Delta t\leq f\cdot {\left[\frac{h}{c}\right]}_{\min}$$
  where Δt is the time increment; *f* is the stability time step factor; *h* is the characteristic dimension of an element and *c* is the local material sound speed in an element. The maximum time step that can be used in explicit time integration is inversely proportional to the sound speed of the material and therefore directionally proportional to the square root of the mass of material in an element [16,17,18,19]:
  (6) $$\Delta t\propto \frac{1}{c}=\frac{1}{\sqrt{\frac{C_{ii}}{\text{ρ}}}}=\sqrt{\frac{m}{VC_{ii}}}$$
  where $C_{ij}$ is the material stiffness (i=1,2,3); ρ is the material density; *m* is the material mass and *V* is the element volume. Artificially increasing the mass of an element can increase the maximum allowable stability time step and reduce the number of time increments required to complete a solution. Mass scaling is applied only to those elements which have a stability time step less than a specified value. If a model contains relatively few small elements, this can be a useful mechanism for reducing the number of time steps required to complete an explicit simulation.

### 5.4. Material Modelling

**Figure 10:**
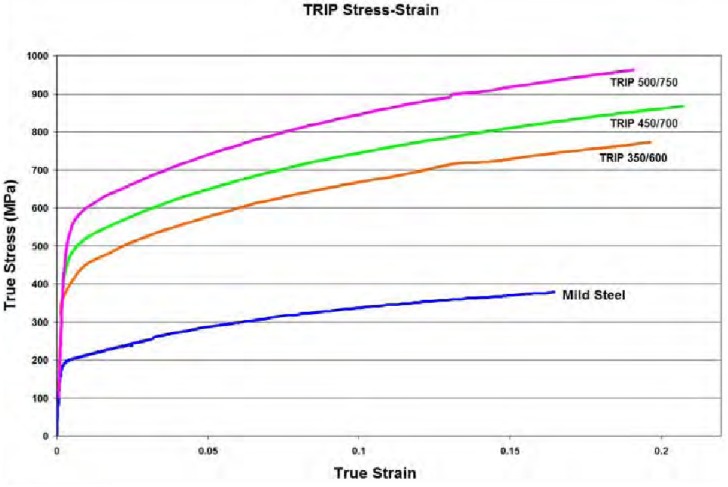
True stress-strain curves for TRIP steel grades.

**Figure 11:**
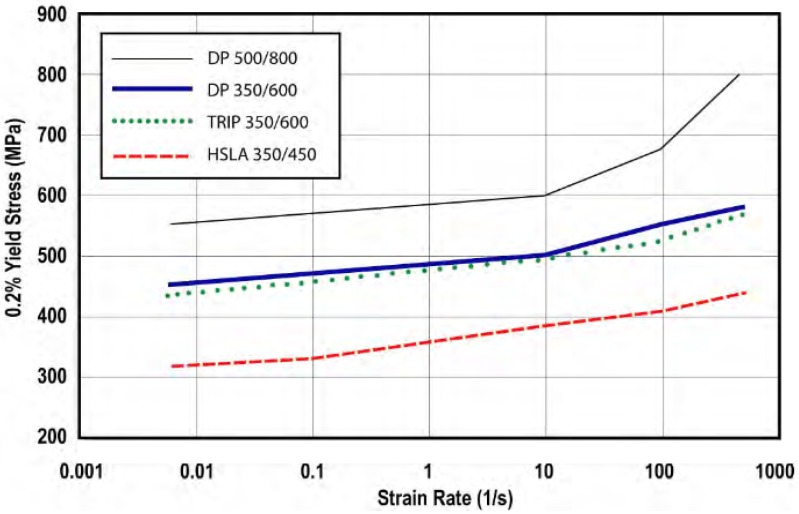
Increase in yield stress as a function of strain rate.

TRIP 350/600 steel (its yield stress is 350 MPa) is employed for the rail components. This kind of steel is commonly used in the automotive industry due to its great strain hardening properties along the plastic zone, which allows good energy absorption under impact. Both the true stress-strain curve (see Figure 10) and the increase in yield stress depending on the strain rate (see Figure 11) are taken from World Auto Steel [21].

**Figure 12:**
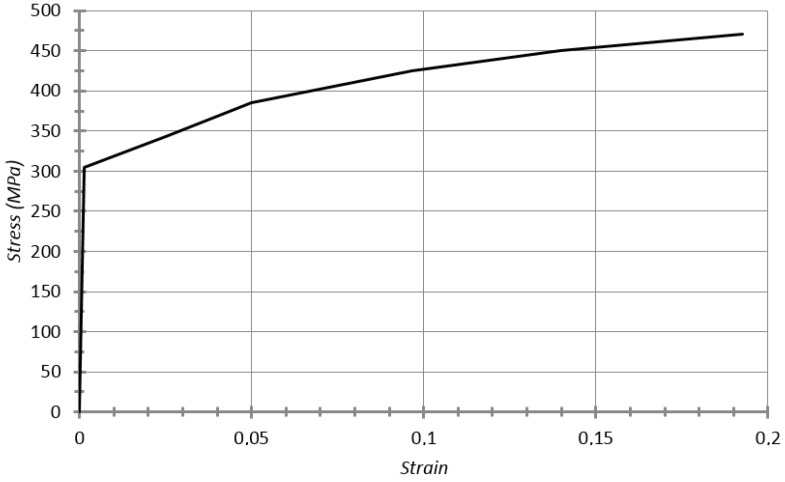
Stress–strain curve for bilinear material TRIP 350-600.

The material is performed with model 024-Piecewise Linear Plasticity, simplifying the deformation behavior into two linear phases (elastic and plastic), as can be seen in the material stress-strain curve (see Figure 12). The real data used to define this simplification is presented below:

- Young’s modulus: 210 × 10^9^ Pa;
- Poisson’s ratio: 0.3;
- Density: 7.850 × 10^3^ kg/m^3^;
- Yield stress: 350 × 10^6^ Pa;
- Tangent modulus: 2.050 × 10^9^ Pa;
- Ultimate plastic strain: 0.2.

Strain rate hardening is given as a table showing the multiplying factor of the yield depending on the strain rate (see Table 1):

**Table 1:** Multiplying factor for yield stress as a function of strain rate.

Strain Rate (s^−1^)	Multiplying Factor for Yield Stress
0.01	1.194
10	1.389
100	1.444
500	1.611

### 5.5. Analysis Settings and Solver

Once the geometry is discretized, the solver (Ls-Dyna) will activate the particular type of element, which in this case is Formulation 16, a fully-integrated SHELL element with 3 through thickness integration points for SHELL elements and constant stress for SOLID elements.

A temporary cut-off is imposed at 25 ms after the release of the system. In this period of time, 10^7^ calculating cycles are performed for the set of 44,000 elements. An error of the maximum energy equal to 10% is defined.

## 6. Results and Analysis

Once the simulation has been carried out, the different outputs are studied. Those with the highest relevance are presented below. In the first place, the energy of system is tackled in order to study the absorption by the different components and finally the behavior at different speeds (specifically 56 km/h and 40 km/h), since they are representative speeds of such vehicles (for example, the EuroNCAP test [13] is performed at 50 km/h).

### 6.1. Energy Absorption by Different Components

One of the objectives of this work is to build a 3D model with real-profile-tubes adapted for quadricycles that achieves the energy absorption distribution proposed by Witteman [9]. That is, 50% of the vehicle energy is absorbed by the set of two front rails, with 7.5% by each crash box, 7.5% by the first portion of each frame rail and 10% by the second part of each frame rail. After an iterative design process, the final geometry is defined and the system is tested with an initial velocity of 15.55 m/s ≈ 56 km/h. The percentages of energy absorption obtained here by FEM numerical simulation are very similar to those proposed by Witteman [9] for conventional vehicles.

**Figure 13:**
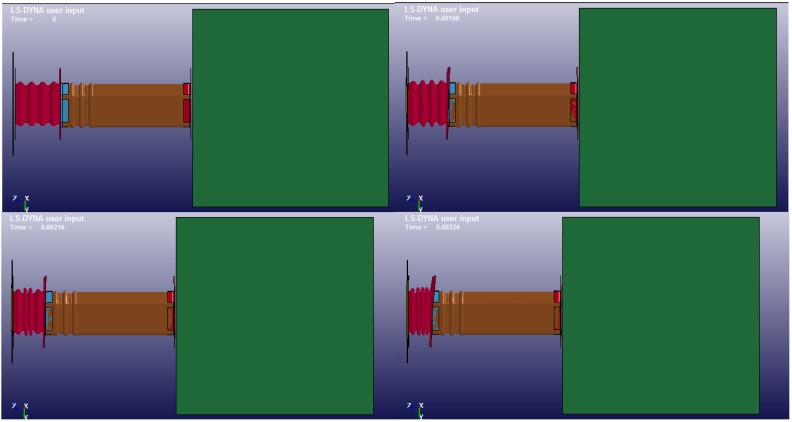

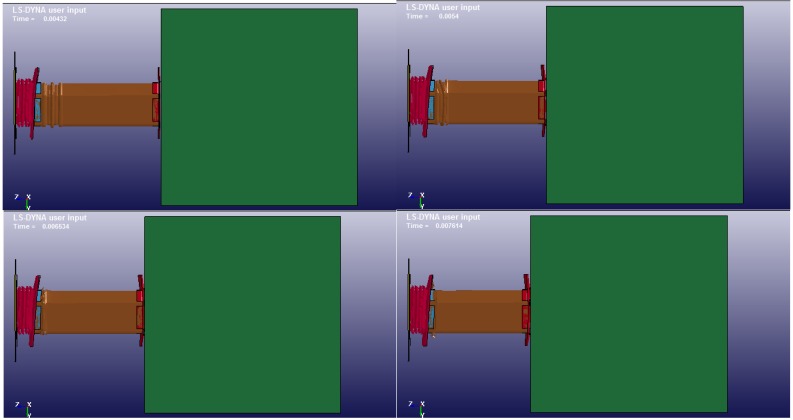
Time evolution of crash box, flanges and frame rail.

The evolution over time of the front rail components can be seen below (see Figure 13). It can be seen that during the first 3.8 ms only the crash box works, that is, it collapses; afterwards, the first part of frame rail (containing ripples) works until 6.2 ms; and finally, the second part of the frame rail comes into action until 11.8 ms. At this time the simulation is cut off, since the engine block is expected to impact, which is not modelled in this system.

**Figure 14:**
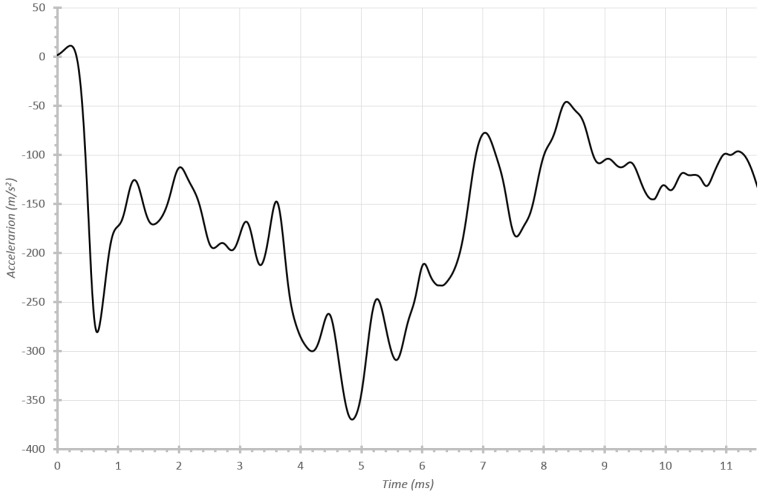
Temporal evolution of acceleration of the ballast with initial impact velocity of 56 km/h.

From the simulation results, the ballast accelerations over time can be obtained (see Figure 14). Here, the different work zones are noticeable: the crash box works at 160 m/s^2^, then the first part of the frame rail at 260 m/s^2^ and finally the second part of the frame rail at 120 m/s^2^, respectively.

In this case, accelerations when the second part of the frame rail collapses are lower due to this part tearing and therefore losing a great deal of rigidity. It must be kept in mind that these rails do not work independently in reality, but as parts of the whole vehicle structure.

**Figure 15:**
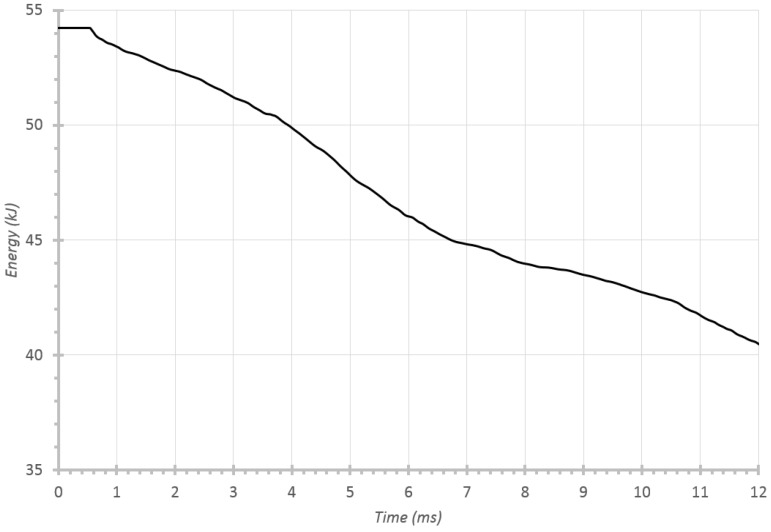
Temporal evolution of ballast kinetic energy with an initial impact velocity of 56 km/h.

If the evolution of the energy absorption over time is studied (see Figure 15), it may be possible to calculate roughly the corresponding amount for each component, *i.e.*, the crash box, and the different parts of the frame rail. Therefore, by taking the intervals 0–3.8 ms, 3.8–6.2 ms and 6.2–11.8 ms as the working times, the percentages of energy absorption for each element can be obtained. By doing this, 7.3%, 7.5% and 10% were found respectively, compared to 7.5%, 7.5% and 10% proposed by Witteman [9]. This result proves that the proposed system faithfully reproduces Witteman’s percentages [9].

Despite the fact that a great amount of kinetic energy remains in the system at the cut-off, it must be taken into account that only the behavior of one rail was reproduced. Therefore, an equivalent whole rail set would absorb twice as much energy, plus the later absorption provided by the engine block.

### 6.2. Behaviour at Different Velocities

**Figure 16:**
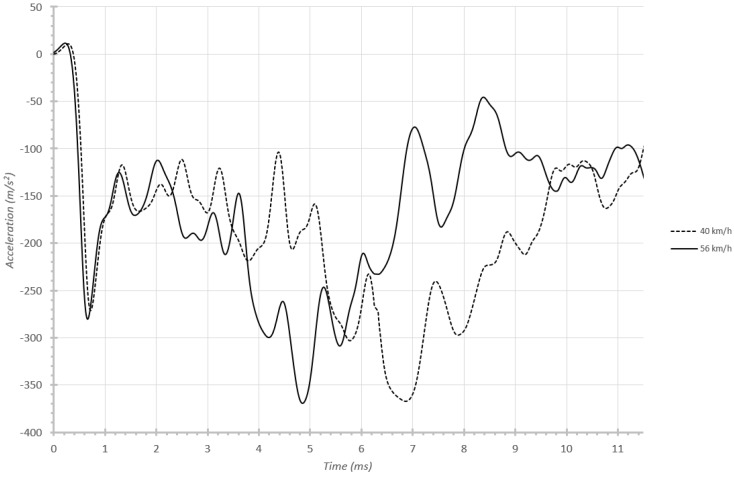
Influence of velocity on ballast temporal acceleration.

Once the design is validated by fulfilling the absorption percentages in a 56 km/h impact, other types of studies can be carried out. For instance, comparison can be made of behavior in 40 km/h and 56 km/h impacts, which is common NCAC [22] practice. By studying this, the relevance of the undulating shaped parts (both the crash box and the initial part of the frame rail) in causing fairly constant acceleration curves becomes clear. In fact, the accelerations obtained with the different velocities are very similar in magnitude (see Figure 16), although with some logical slowing in the 40 km/h case.

## 7. Conclusions

This work proposes an energy absorption system for heavy quadricycles. It includes modifications of the vehicle front rails and the inclusion of attached parts, similar to those in conventional vehicles. The whole front rail set would contain a crash box, flanges and frame rail, all of which are designed to fulfill the energy absorption distribution proposed by Witteman, Santis and Leuwen [9,10,11]. Furthermore, an explicit dynamic calculation methodology is developed to validate the proposed system and study the impact behavior, utilizing Ansys and Ls-Dyna software [16]. Finally, the design proposed achieves very similar absorption percentages (7.3%, 7.4% and 9.8%) to those the authors assign to conventional vehicles. Additionally, before the implantation of this energy absorbing system, it is recommended to carry out experimental tests for a complete verification of this one. Therefore, it is considered that the current poor behavior of quadricycles under impact could be enhanced by the proposed system.
